# Cervical Remodeling/Ripening at Term and Preterm Delivery: The Same Mechanism Initiated by Different Mediators and Different Effector Cells

**DOI:** 10.1371/journal.pone.0026877

**Published:** 2011-11-02

**Authors:** Juan M. Gonzalez, Zhong Dong, Roberto Romero, Guillermina Girardi

**Affiliations:** 1 Perinatology Research Branch, National Institute of Child Health and Human Development (NICHD)/National Institutes of Health (NIH), Bethesda, Maryland, United States of America; 2 York College, City University of New York (CUNY), New York, New York, United States of America; Institute of Zoology, Chinese Academy of Sciences, China

## Abstract

**Background:**

Premature cervical remodeling/ripening is believed to contribute to preterm delivery (PTD), the leading cause of perinatal morbidity and mortality. Despite considerable research, the causes of term and PTD remain unclear, and there is no effective treatment for PTD. We previously demonstrated that complement activation plays a causative role in cervical remodeling that leads to PTD in mice.

**Methodology/Principal Findings:**

Here we found that complement activation is not required for the physiological process that leads to term delivery in mice. Neither increased C3 cervical deposition nor increased C3a and C5a serum levels were observed at term. In addition, macrophages infiltration was found in PTD in contrast to term delivery were no leukocytes were found. Despite the different role of complement and different cellular effector cells, PTD and term delivery share a common dowsntream pathway characterized by increased metalloproteinases (MMPs) release and increased collagen degradation. However, different sources of MMPs were identified. Macrophages are the source of MMPs in PTD while cervical fibroblasts and columnar epithelial cells synthesize MMPs at term delivery. A dramatic diminution in serum progesterone levels precedes parturition at term but not in PTD, suggesting that progesterone withdrawal initiates cervical remodeling at term. On the other hand, MMPs release in PTD is triggered by C5a.

**Conclusion and Significance:**

In conclusion, preterm and term cervical remodeling occur through the same mechanism but they are initiated by different mediators and effector cells. That complement activation is required for PTD but not for the physiological process that leads to term delivery, suggests that complement is a potential specific biomarker and selective target to prevent PTD and thus avert neonatal mortality and morbidity.

## Introduction

The process of parturition has been extensively studied. However, the mechanisms responsible for the onset and progression of labor are multifactorial and not completely understood [1]–[3]. Understanding the mediators and effectors of labor could have enormous benefit, allowing potential interventions for efficient induction of labor or conversely in postponing unwanted labor. Identification of targets to regulate or terminate labor is of particular importance in the treatment and prevention of preterm labor, which continues to be a major public health issue and the number one cause of neonatal death. Among the important events contributing to labor is the ripening/ remodeling of the cervix [4]–[6]. Changes in the cervical structure soften and dilate the cervix allowing the passage of the fetus forced by rhythmic myometrial contractions. We previously demonstrated that complement activation plays a crucial role in the cervical remodeling process that leads to preterm labor [7]. Using a mouse model of PTD induced by LPS we found that complement split product C5a attracts and activates macrophages to the cervix [7]. In response C5a, macrophages release metalloproteinases (MMPs) that degrade collagen, increasing the cervix distensibility and leading to PTD in mice [7].

The aim of this study is to characterize the term cervical ripening process and compare it with preterm. Knowing that complement activation plays a causative role in PTD in mice, we decided to study if complement activation also plays a role in cervical remodeling at term. If complement activation is not involved in the physiological process that leads to term delivery we will then have identified a possible specific and selective target to prevent PTD and thus improve neonatal health.

## Materials and Methods

### Ethics Statement

Our animal studies were performed following the Russell and Burch [8] Three R's (Replacement, Reduction and Refinement) concept to minimize animal use and pain or distress while still achieving the critical scientific objectives. All the experiments performed were conducted in accordance with the National Institute of Health guidelines on laboratory animals and with approval from the Wayne State University (protocol A 09-08-09) and York College CUNY committee on Animal Use and Care (protocol R-3-2009).

### Animals

C57BL/6 timed-pregnant mice were purchased from Jackson Laboratories (Bar Harbor, ME). Animals were shipped on day 10 to 12 after mating. Animals were acclimated in the animal facility for 3 to 5 days before use in these experiments.

Cervical remodeling was studied in mice at term and during preterm labor. A mouse model of PTD which resembles most clinical scenarios in that localized inflammation occurs without systemic maternal illness was used [7]. In this model, mice were treated with LPS (E. coli serotype 055:B5 (250 µg/mouse, intravaginally)) on day 15 of pregnancy. Most of the pregnant females (94.7%) delivered within 24–36 hours of LPS administration [7]. No maternal morbidity or mortality was observed in this model.

Pregnant mice treated with LPS were euthanized antepartum (between 12–18 hours after treatment) or during delivery (intrapartum). Delivery was considered preterm if it occurred within 48 hours after LPS administration (before gestational day 17). In our animal facilities, term delivery occurs between day 20 and day 21 of pregnancy. Control mice were euthanized antepartum (day 17, 18 and 19) or intrapartum (by direct observation after the passage of 1 or 2 pups). Blood samples and cervical tissue were collected.

### Serum complement activation products and progesterone levels

To determine the role of complement activation in term and PTD, generation of complement split products C3a and C5a were measured by measuring the more stable metabolites C3adesArg and C5adesArg in serum. C3a(desArg) and C5a(desARg) result from the removal of the C-terminal arginine by ubiquitous carboxypeptidases and have longer half time than C3a and C5a. C3adesArg in serum was measured by sandwich ELISA as previously described [7] using rat anti-mouse C3a and biotin rat anti-mouse C3a. Both antibodies were purchased from BD Biosciences Pharmingen, CA. A Standard curve was performed using purified mouse C3a protein (native) (BD Biosciences Pharmingen). C5adesArg was also measured by sandwich ELISA using rat anti-mouse C5a and biotin rat anti-mouse C5a [7]. Both antibodies and mouse purified C5a protein for the standard curve were purchased from BD Biosciences.

Progesterone levels were measured by competitive enzyme immune assay (Cayman Chemicals, Ann Arbor, MI).

### Immunohistochemistry and immunocytochemistry

Cervical tissues isolated during antepartum and intrapartum were frozen in O.C.T. compound, and cut into 10 µm sections. Sections were stained for C3 with rabbit anti-mouse C3 (LifeSpan Biosciences, Seattle, WA), neutrophils were stained with rat anti-mouse granulocyte RB6-8C5 mAb (BD Biosciences Pharmingen) and macrophages with F4/80 (Novus Biologicals, Inc, Littleton,CO). MMP-2 and MMP-9 were detected in frozen cervical sections using rabbit polyclonal anti-mouse MMP-2 and MMP-9 antibodies (Abcam, Cambridge, MA). Collagen I distribution was determined using Masson's Trichrome staining and by immunohistochemistry using a polyclonal antibody against collagen type I (ACRIS Antibodies, GmbH, Herford, Germany). HRP-labeled specific secondary antibodies and DAB as substrate were used to develop the respective reactions.

### In situ zymography

Metalloproteinases MMP-2 and MMP-9 activity against collagen I and IV and gelatin were measured by *in situ* zymography as previously described [7], [9]. Briefly, 10-µm-cervix sections were washed in PBS and then incubated for 4 hours with different MMPs DQ substrates. DQ-gelatin, DQ-collagen I and DQ collagen IV (Invitrogen, Carlsbad, CA). The enzyme-driven hydrolysis of these substrates results in an increase in fluorescence signal. Increased fluorescence indicates increased gelatin and collagen degradation by MMPs. In parallel, control sections were preincubated with buffer containing the MMP inhibitor EDTA to indicate the contribution of MMPs [7]. The reaction was stopped by a 10 minutes incubation in 4% paraformaldehyde-PBS. Finally, mounting medium supplemented with DAPI (Vector Laboratories, Burlingame, CA) was applied. Sections (4–6 tissue sections/group) were observed under a fluorescence microscope Nikon Eclipse 50i (Nikon Inc, Melville, NY) and photographs were taken using a Nikon DigiSight Color Digital Camera System and NIS-Elements Research Imaging Software.

### Human cervical epithelial cells

A cell line from normal human endocervical epithelia immortalized by expression of human papillomavirus (Endl/E6E7) (ATCC Manssas, VA) was used in *in vitro* studies [10]. The morphology of these immortalized cells corresponds to glandular tall columnar epithelial cervical cells and closely resembles that of the tissue of origin and primary cultures. Thus, End1/E6E7 cells constitute a reproducible in vitro model to study the role of these epithelial cells in cervical ripening. End1 cells were cultured as previously described [10]. The expression of C5aR on End-1cells was studied by immunohistochemistry using anti-human C5aR antibodies (Cell Sciences, Canton, MA). MMP-2, MMP-9 and progesterone receptors (PR) expression in End-1 cells was also evaluated by RTPCR. Aliquots of End1 cells were incubated for 16 h with progesterone (1 mg/ml) and synthesis of MMP-2 and MMP-9 was studied by RT-PCR.

Aliquots of End1/EGE7 cells were cultured on cell chamber slides incubated with and without progesterone (1 mg/ml) for 16 h and then subjected to in situ zymography using DQ-gelatin and DQ-collagen I as previously described. Immunohistochemistry was performed to detect MMP-2 and MMP-9 using rabbit polyclonal anti-mouse MMP-2 and MMP-9 antibodies (Abcam, Cambridge, MA) followed by a FITC-labelled anti-rabbit antibody (Abcam, Cambridge, MA).

### Quantitative reverse transcriptase-polymerase chain reaction (qRT-PCR)

To determine whether End1 synthesize MMP-2, MMP-9 and PR, qRT-PCR was performed. RNA was harvested from End1 cells with RNeasy Mini Kit (Qiagen, Valencia, CA) and 1 µg of total RNA was reverse transcribed. Primer sequences mouse GAPDH, human MMP-2, MMP-9 and PR were obtained from Applied Byosystems (Foster City, CA). Relative expression was normalized for levels of GAPDH. Relative quantification of MMP-2 and MMP-9 between End-1 cells incubated with and without progesterone gene expression was performed using 2^−DDCT^ data analysis. The comparison was performed in pairs using the same target gene and the same End-1 samples. CT = Threshold value; DCT = average target CT – average GAPDH CT; DDCT (for the same target gene) = average DCT End-1 incubated with progesterone- average DCT End-1 incubated without progesterone. DDCT = 0 when the End-1 of interest are used as calibrator. 2^−DDCT^ = normalized target gene amount (MMP-2 or MMP-9) relative to target gene amount in End-1 cells incubated without progesterone. * p<0.05, n = 4.

## Results

### Serum progesterone levels in term and preterm delivery

**Figure 1:**
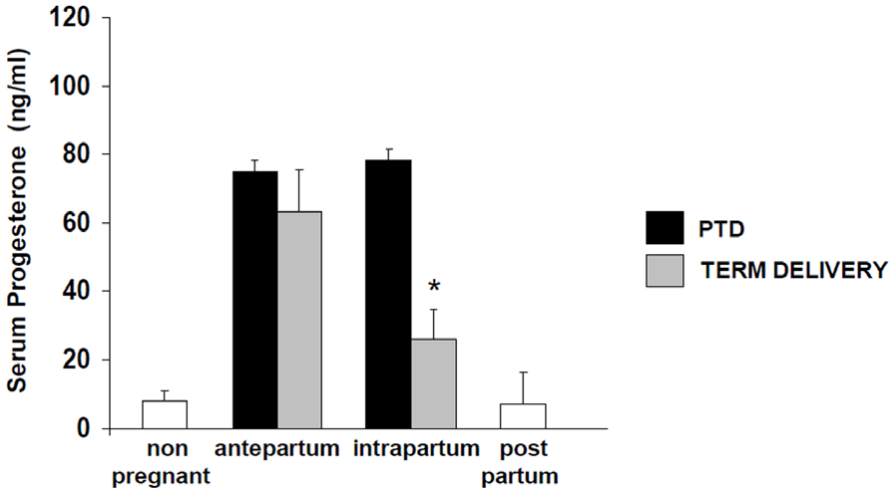
Serum progesterone levels in PTD and term delivery. Antepartum LPS serum samples were collected at day 15 of pregnancy, intrapartum LPS samples at days 16 and 17, antepartum term samples at days 17, 18 and 19 and intrapartum term at days 20 and 21. Note that progesterone levels drop dramatically near parturition time (days 20 and 21) in control mice at term while LPS-treated mice that delivered preterm did not show changes in progesterone levels. * Different from antepartum, P<0.05. 3 to 5 samples were analyzed at each time point.

In most mammalian species aside from the great apes, labor is initiated by a decrease in circulating progesterone levels (progesterone withdrawal) [11]. Indeed, we found that in mice, serum progesterone levels drop dramatically intrapartum when compared to antepartum levels (Fig. 1). On the other hand, mice treated with LPS that deliver preterm do not show a diminution in progesterone levels compared to antepartum levels. Progesterone levels in antepartum were comparable to intrapartum values in LPS-treated mice (Fig. 1), suggesting that progesterone withdrawal is not involved in preterm parturition in LPS-treated mice.

### Complement activation in term and preterm delivery

**Figure 2:**
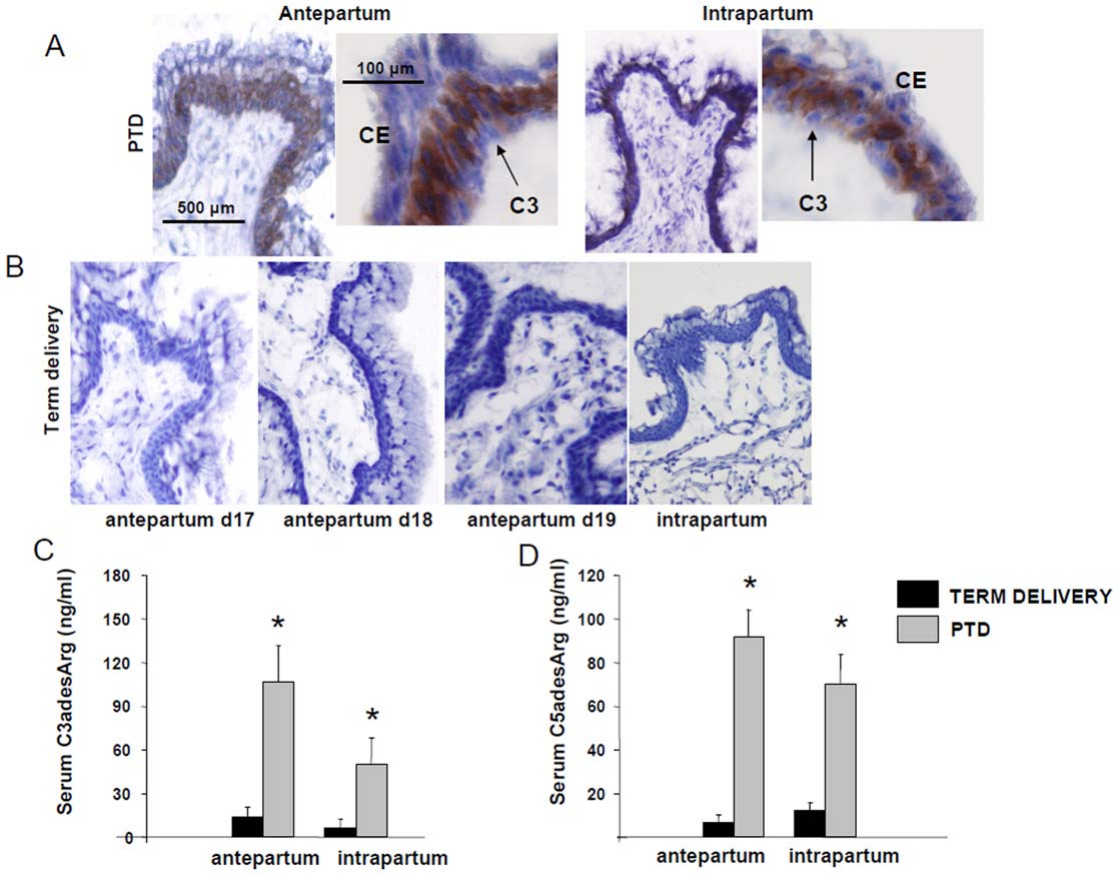
Complement activation in PTD and term delivery. **A-** Complement C3 deposition in the cervix of mice that delivered preterm antepartum and intrapartum. Black arrows point to complement deposition in the cervical columnar l epithelium (CE). Original magnification is ×20. **B-** Complement C3 deposition in the cervix of mice that deliver at term. C3 deposition was not observed antepratum (days 17, 18 and 19) or intrapartum. Original magnification is ×20. For C3 determination, 4 slides per animal were stained and 6–8 animals were studied in each group. **C–D** C3adesArg and C5adesArg levels in serum from mice with PTD and term delivery collected antepartum and intrapartum. Antepartum LPS serum samples were collected at day 15 of pregnancy, intrapartum LPS samples at days 16 and 17, antepartum term samples at days 17, 18 and 19 and intrapartum term at days 20 and 21. * Statistically different from control, p<0.05. n = 5–6 mice/group.

Complement activation involves the cleavage of C3 into C3b that deposits on the tissue, and C3a that can be measured in systemic circulation. Increased C3 deposition was observed in the cervix of LPS-treated mice during antepartum and intrapartum (Fig. 2A). Prompted by the findings that complement activation triggers cervical remodeling and preterm labor (Fig. 2A) [7], we investigated if complement activation also contributes to term labor. Surprisingly, no C3 cervical deposition was observed in mice that delivered at term (Fig. 2B). Negative staining for C3b was observed in cervical samples collected antepartum (days 17, 18 and 19) or intrapartum (Fig. 2B). In addition, mice that delivered at term showed no increase in serum C3a and C5a levels, neither antepartum nor intrapartum (Fig. 2C and 2D). C3a and C5a levels in mice that delivered at term were significantly lower than those observed in mice that delivered preterm (Fig. 2C and 2D). The absence of complement split products at term labor suggests that complement activation is not involve in the physiological process that leads to term parturition.

### Inflammatory cells in term and preterm delivery

**Figure 3:**
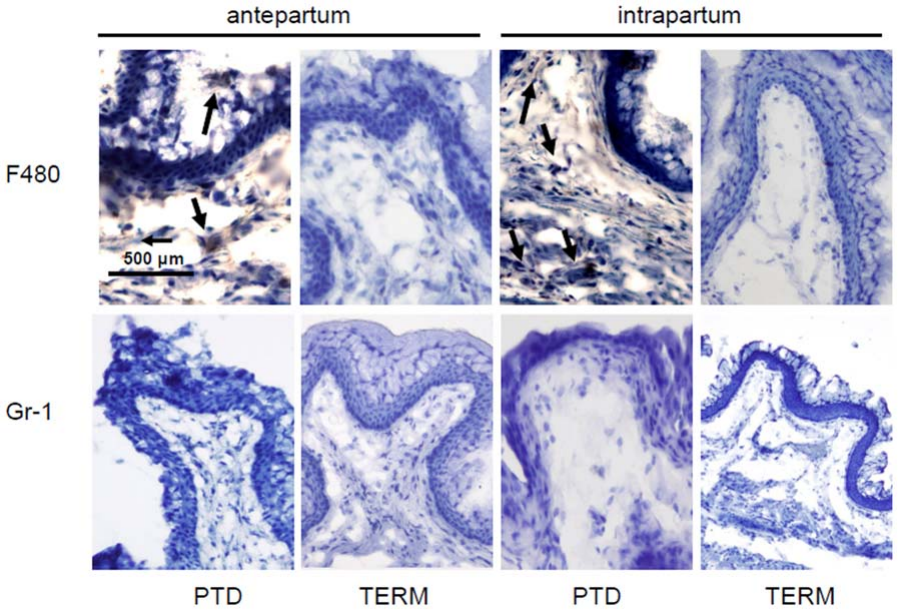
Inflammatory cells in cervical tissue from mice that deliver preterm and at term. **A-** F4/80 staining in cervical tissue. Increased macrophage infiltration (black arrows) was observed in the cervix of mice that deliver preterm antepartum and intrapartum. In contrast no macrophages were observed in the cervix of mice that deliver at term antepartum and intrapartum. Original magnification is ×20. **B-** Gr-1 staining in cervical tissue. No neutrophil infiltration was observed at preterm or term cervical samples. Original magnification is ×20. 4 slides per animal were stained and 6–8 animals were studied in each group.

We previously demonstrated that inflammation plays a crucial role the cervical remodeling that leads to PTD [7]. To determine whether inflammatory cells are also required for the cervical ripening at term, we performed immunohistochemical studies in cervical samples harvested antepartum and intrapartum in control untreated mice. The presence of macrophages was studied using antibodies against F4/80 and neutrophils were identified with antibodies anti-Gr1. Increased macrophages were observed antepartum and intrapartum in the cervix of mice that deliver preterm (Fig. 3) [7]. In contrast, no macrophages were observed in samples collected antepartum and intrapartum at term (Fig. 3).

The absence of inflammatory cells indicates that the cervical remodeling that leads to term labor is not dependent on leukocytes. That inflammatory cells do not participate in the cervical changes that contribute to term labor constitute a significant difference between the term and preterm mechanisms of labor.

Staining with anti-Gr1 showed that neutrophils are not present neither n the cervix of mice at term nor at preterm (Fig. 3) [7].

### MMPs synthesis in human cervical columnar epithelial cells End1

**Figure 4:**
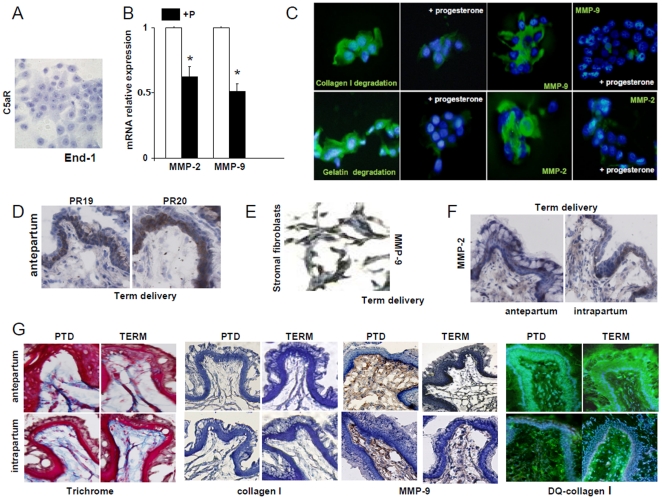
Cervical MMPs activity and collagen degradation at PTD and term delivery. **A-** Immunohistochemical determination of C5aR in End-1 cervical columnar epithelial cells. n = 4. **B–** Relative quantification of MMP-2 and MMP-9 between End-1 cells incubated with and without progesterone. * p<0.05, n = 4 mice/group. Note that in End-1 cells incubated with progesterone a ∼50% reduction in MMP-2 and MMP-9 mRNA was observed when compared to untreated cells. **C-** In situ zymography using substrates DQ-gelatin and DQ-collagen I and immunohistochemical detection of MMP-2 and MM9-9 en End-1 cells incubated with and without progesterone. The green fluorescence indicate the substrate has been digested. MMP-2 and MMP-9 were detected by IHC using specific antibodies followed by FITC-labeled secondary antibodies. D- Immunohistochemical detection of progesterone receptors PR-19 and PR-20 in the cervix of mice during antepartum at term. 4 slides per animal were stained and 6–8 animals were studied in each group. **E-** Trichrome staining, staining for collagen I and for MMP-9 and in situ zymography using DQ-collagen I in cervical tissue harvested antepartum and prepartum from mice that deliver preterm and at term. 3–4 slides per animal and 5 to 6 mice/group were used for immunohistochemical studies. Original magnification is ×20. **F-** Immunohistochemical determination of MMP-9 in cervical stromal fibroblasts at term. 4 slides per animal were stained and 6–8 animals were studied in each group. **G-** Immunohistochemical determination of MMP-2 in cervical columnar epithelial and stromal cells at term during antepartum and intrapartum. 4 slides per animal were stained and 6–8 animals were studied in each group.

MMPs participate in cervical remodeling by degrading the network of collagen fibers and thus increase the distensibility required for delivery [12], [13]. It has been extensively documented that inflammatory cells, macrophages and neutrophils, are a good source of MMPs. The absence of inflammatory cells in the cervical tissue at term leads as to investigate other possible sources of MMPs. In the absence of inflammatory cells, cervical columnar epithelial and stromal fibroblastic cells could be alternative sources of MMPs. To determine whether cervical columnar epithelial cells are capable of synthesizing MMPs, we performed qRT-PCR. MMP-2 and MMP-9 mRNA was found in End-1 cells (Fig. 4B), indicating that cervical columnar epithelial cells can be a source of MMPs in cervical remodeling at term. In situ zymography studies show that End-1 cells produce and release proteases that digest gelatin and collagen I (Fig. 4C). These active proteases were identified as MMP-2 and MMP-9 by immunohistochemistry (Fig. 4C).

Next we investigated the presence of C5aR in End-1 cells. C5aR could not be detected on End-1 cells by immunohistochemistry (Fig. 4A). The absence of C5aR in End-1 cells suggests that cervical columnar epithelial cells are not cellular effectors in PTD, a complement-dependent process. Indeed, we previously described that macrophages rich in C5aR are the main source of MMPs in PTD [7].

In addition, we found that End-1 cells express progesterone receptor (PR) at the mRNA level. Interestingly, progesterone that inhibited MMP release from macrophages in PTD [7] inhibits the synthesis of MMP-2 and MMP-9 in these cells (Fig. 4B). In agreement with these results, we were able to detect progesterone receptors (PR) 19 and 20 in mouse cervical epithelial cells by immunohistochemistry. As shown in Figure 4D, positive staining for PR-19 and PR-20 was observed in the cervical columnar epithelium at term.

We also explored the possibility that cervical fibroblastic cells express MMPs. In accordance to our hypothesis, we found positive staining for MMP-9 in the fibroblasts stroma harvested antepartum in mice that deliver at term (Fig. 4E). Thus, cervical stromal fibroblastic cells can be another source of MMPs leading to cervical remodeling and parturition at term. In addition, we measured MMP-2, another proteolytic enzyme involved in cervical remodeling. We detected MMP-2 in the epithelium and stroma antepartum and intrapartum at term (Fig. 4F). We were not able to detect MMP-2 expression in the cervical tissue of mice that deliver preterm (data not shown).

### Cervical MMPs and collagen degradation in term and preterm delivery

MMPs degrade collagen in the cervix and prepare it for dilation and delivery [12], [13]. Regardless the different sources of MMPs in preterm and term, remodeling of the cervix and massive collagen degradation was observed antepartum and intrapartum in both term and preterm delivery (Fig. 4G). Masson's trichrome (TC) staining revealed a loose array of disordered collagen fibers in the cervix of mice that deliver preterm and term (Fig. 4G). Immunohistochemical studies with specific antibodies against mouse collagen I showed a similar distribution pattern of collagen (Fig. 4G). Collagen packing was disorganized, collagen fibers looked thinner and greater spacing between fibers were observed both antepartum and intrapartum in mice that delivered at term and preterm (Fig. 4G).

Decreased density in collagen fibers was associated with increased expression of MMP-9 (Fig. 4G). In situ zymography using DQ collagen I as substrate revealed an increase in collagenolytic activity in the cervix of mice that deliver at term and preterm (Fig. 4G).

## Discussion

Is cervical remodeling in PTD caused by the same mechanism/s that cause/s cervical remodeling at term but these changes are accelerated in time? This question has been pondered by obstetricians seeking for strategies to prevent PTD for many years. To answer this question we investigated the initiators and cellular effectors in a mouse model of preterm delivery and in control mice that delivered at term. We previously described that complement activation plays a causative role in PTD in mice [7]. Thus, we sought to investigate if complement activation also plays a role in cervical remodeling at term. Here we found that complement activation is not involved in the physiological process that leads to term delivery suggesting that we identified a possible specific and selective target to prevent PTD. In addition, serum complement C3a and C5a levels, that increased during PTD did not increase in mice that deliver at term. That C3a and C5a increase only during PTD suggests that they can be potential biomarkers. This is in agreement with several clinical studies that demonstrate a potential role of complement split products as biomarkers of PTD [14]–[16].

Recent studies in mice suggested that the mechanisms of cervical remodeling in preterm birth is different from normal ripening at term [17], [18]. In one of these studies the expression of proinflammatory genes is upregulated in preterm birth compared to term [17]. The other study demonstrated that cervical ripening preterm can be initiated by more than one mechanism and it is not necessarily an acceleration of the physiological process at term [18].

Our data suggest that the cervical ripening at term is a process non-leukocyte dependent. We did not find inflammatory cells in the cervix of mice with term parturition in contrast to mice that delivered preterm [7]. Several animal studies support our observation that cervical remodeling at term is leukocyte-independent. Steroid 5alpha-reductase type 1 null mice (Srd5a1−/−) that do not recruit inflammatory cells in the cervix give birth normally suggesting that inflammatory cells are not required for term parturition [19]. In addition, granulocyte depletion with monoclonal antibodies LyG (Gr1) before birth has not effect on the timing or outcome of parturition in mice [19]. A human study emphasizes the lack of correlation between inflammation and cervical remodeling at term. In this study, the authors reported that inflammation-related genes did not emerge as differentially express with cervical ripening [20]. Even though several studies in animals suggest that inflammatory cells do not participate in cervical ripening at term, some human studies suggest that inflammatory cells do participate in the cervical changes in term parturition [21], [22]. This discrepancy might be caused by the fact that when the biopsies are obtained; intrapartum may overlap with postpartum and the cervical repair process might have already started. Indeed many studies demonstrate the crucial role of inflammatory cells in the cervical tissue repair after delivery [19].

We previously demonstrated that complement component C5a recruits and activates macrophages in cervical tissue leading to cervical ripening and PTD in LPS-treated mice [7]. The absence of complement activation and generation of C5a in cervical tissue at term might explain the absence of inflammatory cells. Despite the absence of complement activation and inflammatory cells at term, our data demonstrate the presence of MMPs and concomitant collagen degradation in the cervix of mice that deliver at term and preterm. Increased expression of MMP-9, disorganized collagen packing, thinner collagen fibers and greater spacing between fibers were observed in both term and preterm. This suggests the existence of a common downstream pathway shared by term and preterm cervical remodeling.

The identification of this common mechanism raises the question as to which cells are responsible for MMP release during cervical ripening at term. It has been established that cervical fibroblasts obtained from pregnant women are capable of releasing MMPs in culture [23]. Other studies also showed that human cervical fibroblasts can also synthesize MMPs [24], [25]. In addition, Imada and collaborators demonstrated that cervical fibroblast from rabbits synthesize MMP-9 in culture [24]. Indeed, we found positive staining for MMP-9 in cervical fibroblastic cells in the stroma of control mice at term.

Staining for MMP-9 and robust activity against collagen I was also observed in the cervical columnar epithelium in mice at term. Thus, we performed in vitro studies using End-1 cells [10] (human columnar epithelial cells) to corroborate that these cells can indeed synthesize and express MMPs. qRT-PCR studies revealed the presence of MMP-2 and MMP-9 mRNA and in situ zymography and immunohistochemistry studies demonstrate the presence of active MMP-2 and MMP-9 in End-1 cells, suggesting that columnar epithelial cells can be the source of MMPs responsible for cervical remodeling that leads to parturition at term.

We also need to consider that cytokines secreted by the myometrium, placenta and fetal membranes at term contribute to the release of MMPs in the cervix in the absence of inflammatory cells [26], [27]. Thus, it is possible that inflammatory mediators secreted by neutrophils infiltrating the fetomaternal interface reach the cervical stroma and promote MMPs release.

It has been described that progesterone inhibits the release of MMPs and collagenolysis [28], [29]. Interestingly, we found that progesterone inhibits the synthesis and expression of MMP-2 and MMP-9 in End-1 cells. That progesterone inhibits MMPs release suggests that a diminution in progesterone levels might trigger the release of MMPs and induce cervical ripening at term. Indeed, we found that progesterone levels drop dramatically in mice around parturition time at term, in contrast to mice that deliver preterm. This observation suggests that progesterone withdrawal can trigger MMPs release and subsequent parturition at term as has been suggested [11]. We were not able to detect C5aR on End-1 cells, suggesting that complement activation that is responsible for the release of MMP-9 from macrophages in PTD is not involved in MMP release from cervical columnar epithelial cells. Thus, we propose that progesterone withdrawal triggers MMPs release and parturition at term. That progesterone receptors PR-19 and PR-20 are present in cervical epithelial cells reinforces this concept.

**Figure 5:**
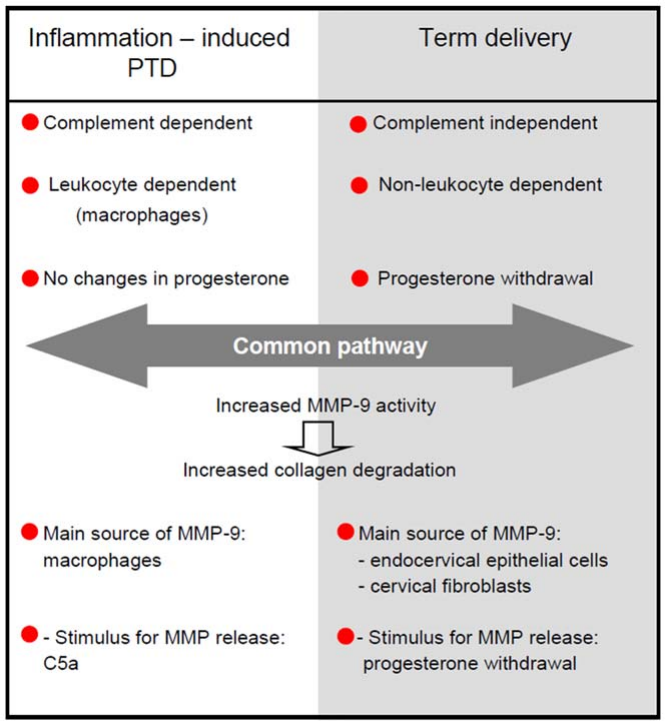
Distinct and similar features during preterm and term cervical remodeling. PTD is initiated by complement activation while complement activation is not required for term parturition. In addition term delivery was found to be non-leukocyte dependent while on the other hand preterm delivery is mediated by macrophages. A dramatic diminution in serum progesterone levels precedes parturition at term but no progesterone levels changes are observed in PTD. PTD and term delivery share a common dowsntream pathway characterized by increased MMPs release and increased collagen degradation. The sources of MMPs in PTD are macrophages while cervical stromal fibroblasts and columnar epithelial cells seem to be the source of MMPs at term. Anaphylotoxin C5a is the trigger for MMPs release in PTD while progesterone withdrawal seems to be the trigger at term. In conclusion, preterm and term cervical remodeling occur through the same mechanism but it is initiated by different triggers and effector cells.

In Figure 5 we summarize the distinct and similar features that characterize cervical remodeling at preterm and term. 1) Preterm delivery is initiated by complement activation while complement activation is not required for term parturition. 2) Term delivery was found to be non-leukocyte dependent while on the other hand PTD is mediated by macrophages. 3) Macrophages are the source of MMPs in PTD while cervical stromal fibroblasts and columnar epithelial cells seem to be responsible for the production of MMPs in cervical remodeling at term. 4) The trigger for MMPs release is C5a in PTD while our studies and other studies [11] suggested that progesterone withdrawal is the initiator of cervical ripening at term.

Regardless the different triggers and cellular mediators, we identified a common downstream pathway that involves MMPs release and collagen degradation in both term and PTD.

In conclusion, preterm and term cervical remodeling occur through the same mechanism but they are initiated by different triggers and effector cells. Thus, we demonstrated that preterm birth is not an acceleration of the normal physiological cervical processes that lead to term parturition.

This is the first study to identify the complement system as a potential biomarker and possible specific and selective target for therapy in PTD.
